# VentQsys: Low-cost open IoT system for $CO_2$ monitoring in classrooms

**DOI:** 10.1007/s11276-021-02799-5

**Published:** 2021-10-18

**Authors:** Rafael Fayos-Jordan, Jaume Segura-Garcia, Antonio Soriano-Asensi, Santiago Felici-Castell, Jose M. Felisi, Jose M. Alcaraz-Calero

**Affiliations:** 1 Computer Science Department, Escola Tècnica Superior d’Enginyeria, Universitat de València, Burjassot, 46100 Spain; 2 G-Agua (Tecnologia de la Gestion del Agua), SNLE, Riba-roja de Túria, 46190 Spain; 3 School of Computing, Engineering and Physical Sciences, University of the West of Scotland, PA1 1LU Paisley, United Kingdom

**Keywords:** IoT, WSN, Spatial statistics, $CO_2$, Open-source, Sustainability

## Abstract

In educational context, a source of nuisance for students is carbon dioxide ($CO_2$) concentration due to closed rooms and lack of ventilation or circulatory air. Also, in the pandemic context, ventilation in indoor environments has been proven as a good tool to control the COVID-19 infections. In this work, it is presented a low cost IoT-based open-hardware and open-software monitoring system to control ventilation, by measuring carbon dioxide ($CO_2$), temperature and relative humidity. This system provides also support for automatic updating, auto-self calibration and adds some Cloud and Edge offloading of computational features for mapping functionalities. From the tests carried out, it is observed a good performance in terms of functionality, battery durability, compared to other measuring devices, more expensive than our proposal.

**Supplementary Information:**

The online version contains supplementary material available at 10.1007/s11276-021-02799-5.

## Introduction

Carbon dioxide ($CO_2$) is a colorless and odorless gas. It is naturally found in ambient air in concentrations ranging from 300 ppm to 550 ppm, depending on whether we measure in rural or urban environments. It is produced by (human and animal) breathing and burning fossil fuels. In the atmosphere, this gas produces the displacement of oxygen and in high concentrations (over 30,000 ppm), it can produce rapid breathing, confusion and asphyxiation, by reducing the oxygen concentration below 20% [7]. Actually $CO_2$ is a great indicator of air quality, since it acts as a whistle blower for air renewal. It is known that from concentrations of more than 800 ppm in working environments, complaints due to odors begin to occur. However, the usual levels that we can find in an indoor environment will be related to different variables that affect this factor, such as outdoor air levels, indoor sources, occupancy levels and ventilation rates.

In addition, in the educational sector, high $CO_2$ concentrations are a source of nuisance for students, due to closed rooms, lack of ventilation or circulatory air in classrooms [24] and they can affect the performance of the students. The educational authorities show some concerns, because with this situation not only the performance is compromised but also their health. Several studies have shown some relationship between the environmental pollution levels, in particular those related to $CO_2$, and some breathing diseases: hypercapnia, Chronic Obstructive Pulmonary Disease (COPD), etc. [7, 11].

Although there are some sensors able to monitor $CO_2$ in indoor environments, their prices range from 100 to 300, and their networking abilities are limited to collect data (they only allow download data in csv or pdf format in the measurement device). In this work, our goal is to develop a configurable low-cost open-hardware and open-software IoT system (with nodes costing less than 70) to measure $CO_2$ concentration by using a Non-Dispersive Infrared Absorption Spectroscopy (NDIR) sensor, measuring *T* and *RH* as well. Also, we aim to improve the performance of the system by adding different functionalities for upgrading [20], auto-self networked calibration, as well as include some computional offloading capabilities to Edge and Cloud for advance mapping functions.

This paper is structured as follows. First this introductory section has introduced the problem, explaining the context, the goal and after a section with some related works. Then, the materials and methods section explains the design and implementation of the node, as well the architecture of the IoT system to interconnect these nodes with external tools for control and management. The following section explains the measurement sessions done in different environments and the energy consumption performance evaluation done with the sensing node, discussing also these results. Finally, the conclusions section explains the lessons learnt and summarizes the innovations introduced.

## Related work

In [28], authors measure $CO_2$ concentration in different schools in Serbia. Their measurements exceed recommended concentrations, as over 800 ppm can generate sick building syndrome [27]. This syndrome is described associated to different symptoms as: headache; eye, nose, or throat irritation; dry cough; dry or itchy skin; dizziness and nausea; difficulty in concentrating; fatigue; and sensitivity to odors.

In [16], authors found a correlation between the SARS virus spread and the sick building syndrome. Also in the SARS-COV-2 pandemic context, an increasing concern is raising about the virus spread, related to the fact that the probability of infection increases in indoor environments. This probability is proportional to the $CO_2$ concentration [3, 15] and it is inversely related to the amount of ventilation. As ventilation is the air renovation, i.e. the exchange of potentially contaminated indoor air with outdoor air (theoretically free of virus), it allows the elimination of particles in suspension, which potentially can contain virus, and on its possible pathways [4]. This concern has evolved into recommendations, given by some Governments and research institutions [13, 18], for ventilation in school classrooms and indoor public places in order to reduce the probability of COVID-19 infections. This assertion is supported by [30], where the author concludes that indoor air quality control strategies can be integrated to reduce the risk of SARS-COV-2 infection.

The state of the art in environmental pollution issues is wide and many applications related to Wireless Sensor Networks (WSN) have been deployed. In [19], a survey on different applications of these networks is shown for real-time ambient air pollution monitoring and air quality in metropolitan areas. It must be noticed that these WSNs have been applied in different scenarios during the last two decades for different issues, such as lightning strike detection [17], or soundscape monitoring [21], etc.

It is worth mentioning that among the different aspects involved in this kind of systems for air quality monitoring, both the sensing part and the networking part are the predominant ones. On one hand, focusing on the sensing part, in [9], the authors carry out a performance evaluation of a number of low-cost consumer grade monitors and single-parameter sensors in detecting five indoor environmental parameters: particulate matter (PM or particle pollution), $CO_2$, Total Volatile Organic Compounds (TVOC), dry-bulb air (*T*) and Relative Humidity (*RH*). Their study shows that technological advancements have raised an opportunity for more effective indoor air quality control and management, suggesting that most of the tested monitors have the potential to be used to secure adequate indoor environments by triggering the right chain of actions.On the other hand, focusing on the networking part for environmental monitoring, in [5] are shown optimal WSN deployment models for air pollution monitoring. In [22], the authors describe a system for air quality monitoring in different cities. In this case, the authors, use ThingSpeak to collect data in the Cloud, using different communication technologies in the WSN (i.e. Zigbee, Wifi, LTE and BLE). Also in [14], the authors develop a client-server system with LTE communicaton and with a set of sensors for measuring PM2.5 and PM10 (PM2007), VOCs, CO, $CO_2$, temperature/humidity.

In [23], the authors develop a system for air quality monitoring and temperature (*T*) in classrooms, based on Z-Wave technology sending information to a central gateway. They study the well-being of pupils as it depends on indoor environmental quality and thermal comfort. Finally, in [2], the authors focus on Cloud computing and artificial intelligence developments in order to assist in the air monitoring process. In Table 1, we have summarized some qualitative features comparing our solution with other solutions from the state-of-the-art.

**Table 1:** Qualitative comparison of the proposed solution with other solutions

Work	Sensors	Technology	OTA update	Openness
[5]	Air pollution	WiFi	No	No
[9]	Particulate matter, CO2,	WiFi	No	No
	TVOC, Air Temp and Rel.Hum			
[14]	PM2.5/PM10, VOCs, CO, CO2	LTE	No	No
[19]	Electrochemical (CO2, NO2,	WiFi	No	No
	SO2, PM, NH3 and toxic gases)			
[22]	Air pollution	Zigbee, WiFi,	No	No
		LTE and BLE		
Our proposal	CO2, Temp, Rel. Hum	WiFi	Yes	Yes

## Materials and methods

In this section, the materials and methods used in the development of this IoT system are explained, as well as the architecture used.

### Node development

In the development of the sensing nodes, NodeMCU, a ESP8266-based platform, is used [20]. The NodeMCU is in charge of sampling and reading the $CO_2$ sensor outputs via UART communications with the selected $CO_2$ sensor, and of sending such values using WiFi-based communications. Two possible NodeMCUs models are supported in our prototype: Amica (with ESP-12E and chipset CP2102)[12] and Lolin (with ESP-12F and chipset CH340). The node is completed with a RGB led with common catode to show directly the different levels in the next scale: low or green for values up to 800ppm, medium or blue for values between 800 and 1500ppm, and finally high or red for values greater than 1500ppm. Besides an OLED (organic light-emitting diode) screen shows the values of the different parameters.

**Fig. 1:**
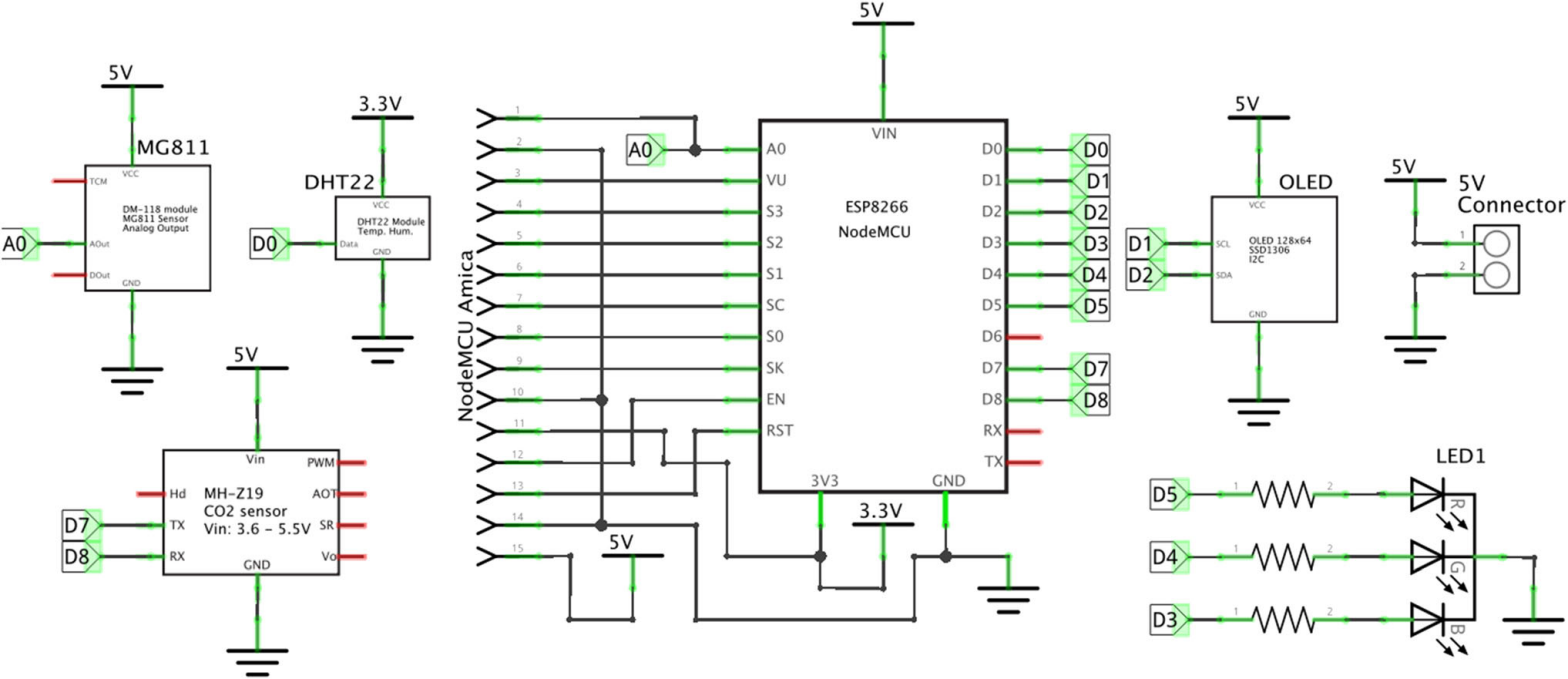
Electronic schematic of the $CO_2$, *T*, *RH* node

**Fig. 2:**
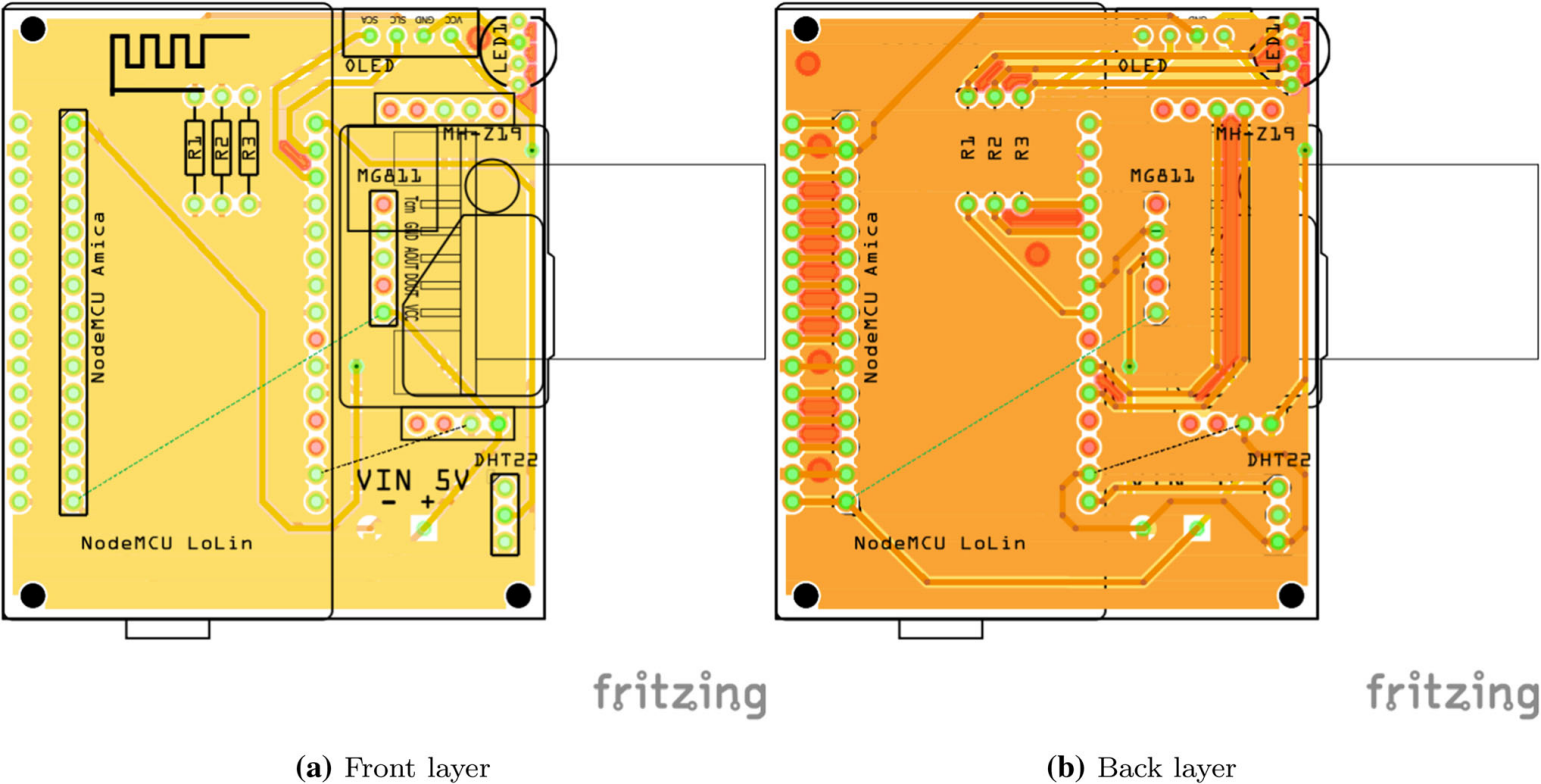
Layers of the PCB

Our proposal is oriented to measure $CO_2$, *T* and *RH*. We have used the so-called NDIR sensor, which is the most popular tool for $CO_2$ monitoring, that does not require analytical grade concentration readings. A NDIR sensor is a simple spectroscopic sensor based on an infrared source (lamp) with a sample chamber (or light tube), a light filter and an infrared detector, providing high accuracy. For this reason, our selection has been made separately with a MH-Z19 [31] (or optionally, MG811 which is based on solid electrolyte cell principle) and a DHT22 [1] (with an accuracy of 0.5$^{\circ }$C for *T*, ranging from −40$^{\circ }$C to 80$^{\circ }$C, and 2% for *RH*, ranging from 0 to 99.9%). In particular, the MH-Z19 sensor has a response time in less than 60 seconds with an accuracy ± (50 ppm + 5% value) in a range from 0 to 5000 ppm. Figure 1 shows the schematics of the whole node where the reader can see the interconnection between the key components. Also Fig. 2 shows both layers of the designed PCB.

**Fig. 3:**
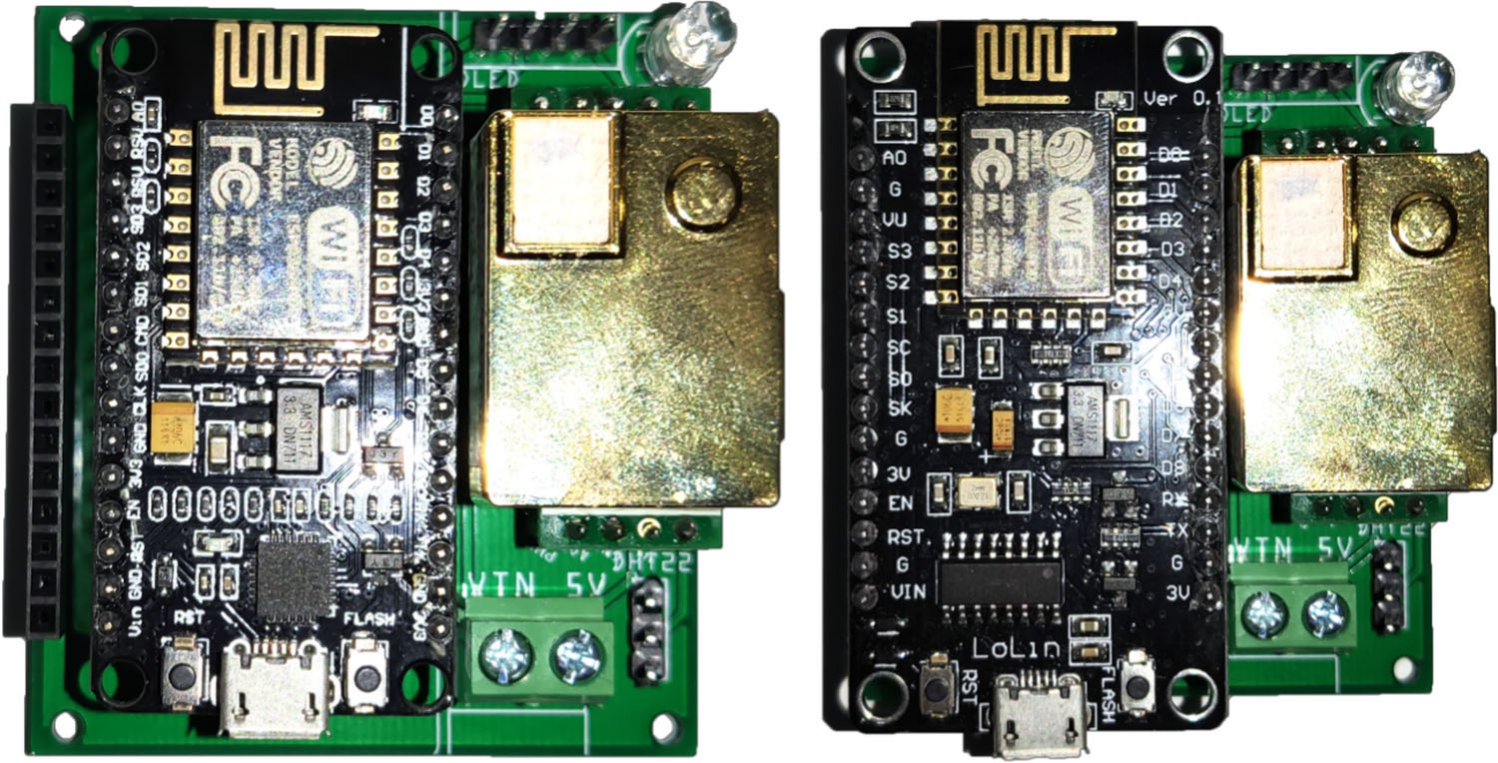
Photograph of the $CO_2$, *T* and *RH* node with Amica and Lolin optional MCU

Such schematics has been layout in a PCB as part of the prototyping of the device. Figure 3 shows a photo of the node using the both mentioned MCUs: Amica with CP2102 chipset and Lolin with CH340 chipset. These nodes are connected via WiFi and send the information to the collection system via REST API.

#### Calibration

NDIR sensors rely on an infrared light source and detector to measure the number of $CO_2$ molecules. But with aging, both the light source and the detector deteriorate, resulting in slightly lower $CO_2$ molecule counts, producing a drift in the readings. MH-Z19 sensor provides different options for calibration: a) auto-calibration according to the background, b) manual calibration referring to 400ppm, usually in a nitrogen environment, and c) digital zero-calibration (also referring to 400ppm), using fresh air. These calibration options have different pros and cons. In this case, the simplest one is the first one (a), based on the background, at the cost of a lower accuracy (around 50ppm plus 5% of the measurement value) that is a negligible amount in our case. This method is based on the fact that in a common environment, $CO_2$ levels come back to 400ppm when there is no $CO_2$ production for few hours, when the classroom is empty or during the night. That is because when there is nobody for a period of time of several hours, $CO_2$ levels drop to a minimum. Also, we have considered option (c) or manual calibration, in which the node is located in an environment with very low levels of $CO_2$, such as open spaces, far away from contamination, taking this reference as 400ppm. For this last option (c), optionally, the nodes have been designed to work with batteries, enabling the movement to suitable places to perform this calibration.

Finally it is worth mentioning that to combat the sensor drifts, during calibration of the sensor, multiple readings are taken. Then an average of these readings is calculated and the difference (or offset) between the new reading and the original reading when the sensor was originally calibrated at the factory is stored in EPROM memory. Thus, this“offset”value is then automatically added or subtracted to later readings. This calibration is made via software.

**Fig. 4:**
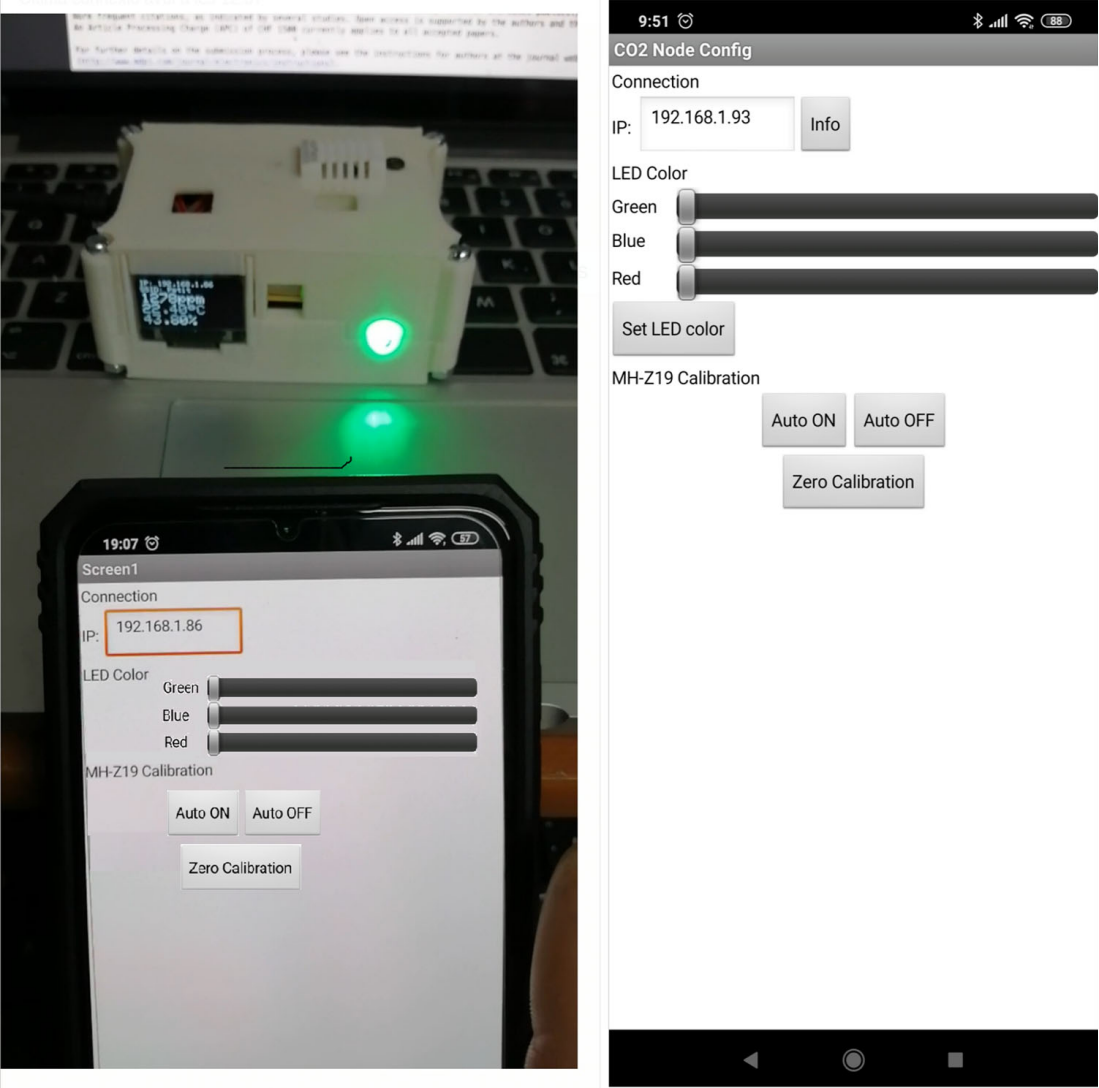
Photograph with the APP for calibration and led control node

In summary, our nodes combine a powering system, based on batteries (4xAA Energizer Max), and a software library to control the calibration options available for our NDIR $CO_2$ sensor. Figure 4 shows a photograph with a mobile App deployed with the calibration functions programmed for this purpose. This photo was done while calibrating one node. For the calibration procedure, we need to connect the APP to the node and then set the low level (p.e. 400 ppm).

### Architecture of the system

**Fig. 5:**
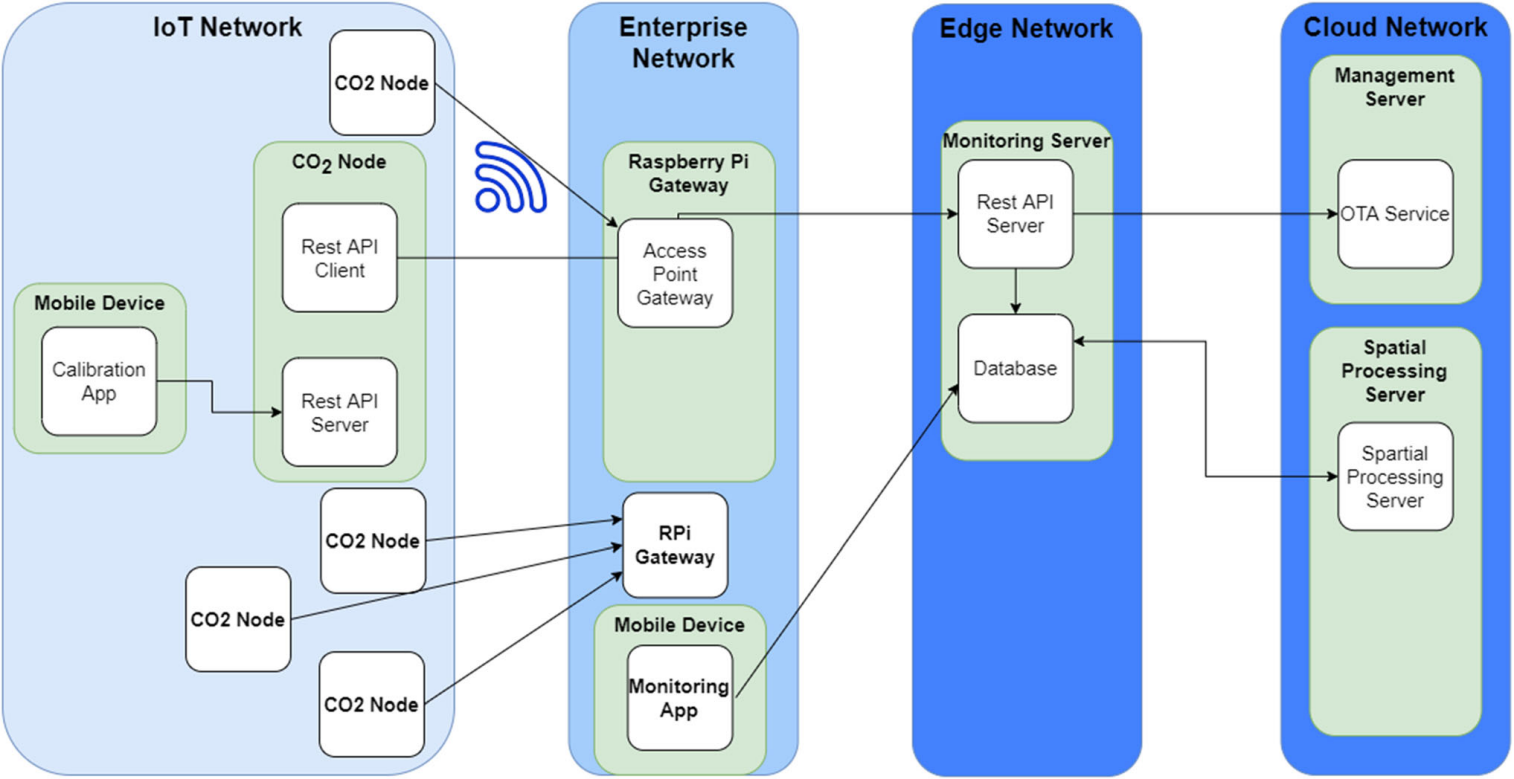
Schema of the system architecture

Figure 5 shows the overall system architecture proposed. The IoT Network is the segment where all the IoT nodes are deployed, in our case the classroom or place being monitored. Each IoT node is exposing an HTTP Server Rest API, used to perform the calibration of the sensors using our mobile application previously described. The IoT node has also a REST HTTP Client used to perform a periodic submission of the monitored information. The IoT nodes are connecting to a RPi via WiFi, sending information of $CO_2$, *T* and *RH*. The RPis are deployed in the enterprise network segment acting as a gateway between the IoT network and the Internet. Notice that the RPis are considered optional devices in our infrastructure. They have been intentionally included to allow IoT devices to be deployed in the desired locations without the requirement to have direct Internet Wi-Fi coverage. However, if this limitation is acceptable for the concrete deployments (use case), the IoT devices are also capable to be directly connected to the Internet. The RPi are able to act as a gateway for a significant number of IoT devices allowing a high-dense deployment. Our experiments have successfully achieved 40 IoT devices simultaneously connected to just one RPi.

In the Internet, we are making use of a mobile edge computing architecture. Such architecture is composed by at least two different network segments worth to be explained. The Edge and Core Network segment. The Edge segment, located close to the Enterprise network segment is where we have decided to perform the deployment of our Monitoring Server. The server is exposing a Server Rest API used by all the IoT devices to upload the sensed information. Such API is in charge of storing the monitored information into a MySQL Database. The same Server Rest API can be used to retrieve the information from a PC or smart phones application. In addition, this information can be used as an input to a control application towards the automation of the ventilation system via machine-to-machine communications. In the most simple scenario, this monitoring server can be located in a stand-alone server, however to deal with scalability we recommend to shift such monitoring server to the cloud and if after this we need to deal with even large scalability, when then shift to edge computing replicating the Server API and keeping load balancing and high performance optimizations for the database.

Once the information is collected from all the IoT devices, we have also developed an application that retrieved such information, allowing to show on real-time the monitored metrics. This application is currently developed by Android and has been installed in our classrooms to allow inhabitants to see the current status. The system is open-source and as such, further visualization tools can be developed to adapt to different execution environments (web, Windows, Linux, Apple Watch, iOS, etc).

As an added-value service, our architecture has another compute-intensive optional component, the Spatial Processing Server. This server allow us to perform spatial interpolation to allow us to estimate the level of concentration of $CO_2$ in every single point of the room by applying advance statistics, described in next subsection. This spatial processing server make use of the data gathered from all the IoT nodes and produces as an output the estimated interpolated value of the metrics for every of the positions of the room. Another optional added-value service compatible with our architecture is the usage of a Over-the-Air (OTA) firmware update server. This service allows us to perform the dynamic update of the firmware for all the IoT devices just in case more functionalities are pushed to the IoT devices without the need to re-deploy them. These additional compute-intensive added-value services are deployed in the Cloud to deal with scalability and compute-intensive requirements for large-scale deployments. It is worth to mention that the proposed architecture ranges from a very simple, client-server (2-layer) architecture until a 4-layer architecture able to deal with very large-scale deployments.

### Spatial statistics

We have also studied the spatial statistical behavior of the information collected. To this end, we have chosen Kriging technique as a spatial interpolation method. The information from the $CO_2$ concentration samples establish a data set based on measurement from different and specific locations. By denoting the determined value of the $CO_2$ concentration measurements at a location *x* as *C*(*x*), this data set is defined as $\{C(x),\,x\in \mathcal {D}\}$, where $\mathcal {D}$ are all the locations of the modelling sets, following the Kriging technique [8].

The proposed model aims to forecast the value $C(x_0)$ in any location $x_0$, specifically those in the validation set. The measurement reports contain information about the set of covariables included. Therefore in (1) , *C*(*x*) is modeled as an average of each covariable involved in the process in the geographical area considered, plus some bounded spatial variability, which is explained by the short term process with spatial dependence.

1 $$\begin{aligned} C(x) = \mu (x) + \delta (x), \end{aligned}$$

where $\mu (x) = \mathbb {E}[C(x)]$ and $\delta (x)$ is a stationary Gaussian process with zero mean, whose spatial dependence characterization is given by the variogram $\gamma$ in (2). [10].

2 $$\begin{aligned} 2\gamma (h) = \mathrm {Var}\left[ C(x+h) - C(x)\right] = \mathrm {Var}\left[ \delta (x+h)-\delta (x)\right] , \end{aligned}$$

where $\mathrm {Var}$ denotes the variance and *h* is an offset.

### Public release

All the software and hardware presented in this paper has been released as open source available at https://github.com/ETSE-UV/VentQ. It includes, the hardware design and schematics together with the complete firmware of the IoT devices, the calibration application for the mobile application as well as the monitoring application to see the gathered metrics on real-time. The code of the monitoring server as well as the spatial processing server has been also released. For the OTA server, we are using an already existing ArduinoOTA open software^1^. The main intention is to make publicly accessible this low cost design to help controlling $CO_2$ concentrations. This is a true open-software and open-hardware design.

## Results and discussion

The evaluation of the performance of our system has been done by measuring in different scenarios, with a particular interest in classrooms during examination periods. Besides, we have evaluated the energy consumption of the nodes, in order to guarantee the life of the batteries, at least during 7 or 8 hours, approximately the duration of an exam including time intervals both at the beginning and at the end.

### Measurements in daily situations

Prior to perform the real tests in classrooms during exam period and in order to check the proper operation of the nodes, we decided to perform different tests during daily activities, such as monitoring $CO_2$ concentration in an office and in a bedroom. Notice that these two scenarios will remain with closed doors during the day.

**Fig. 6:**
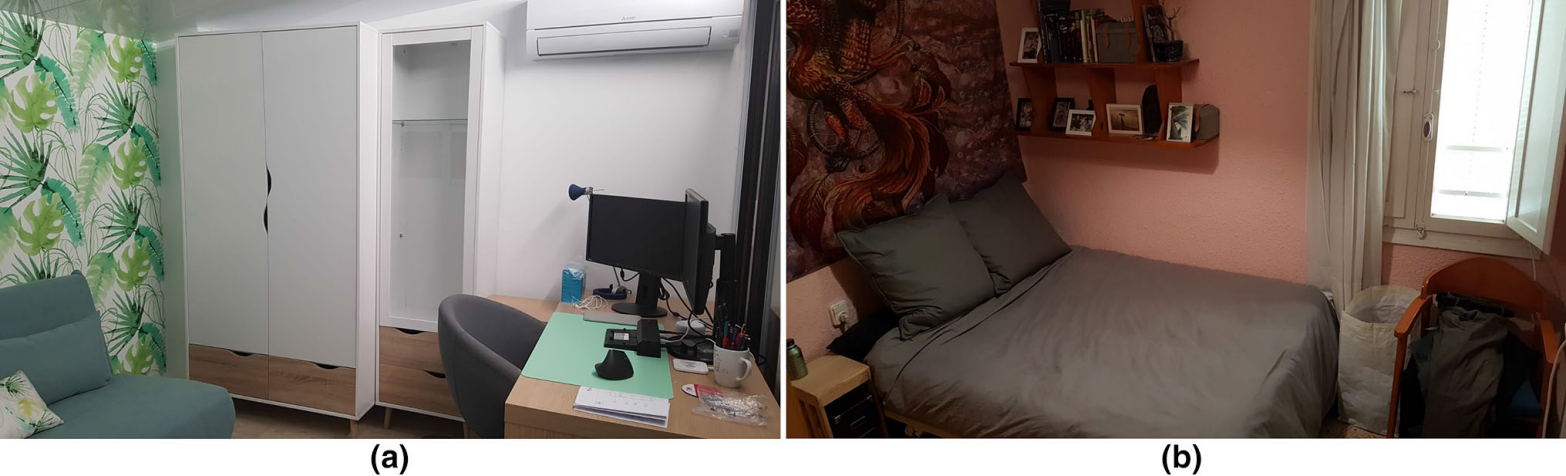
Photograph of the daily scenarios analyzed

Figure 6 a shows an office of approximately 19.8 $m^3$, used at least 10 hours a day by one person, turning on the heating system (by air conditioning) set to 25$^{\circ }$C and turning it off at the end, keeping the room closed the whole time.The bedroom, shown in Fig. 6b, of 26.25 $m^3$, is only used for sleeping at night, by one person, turning on an electric radiator for a few minutes before going bed. Later, it remains closed the whole time, except early in the mornings when the window is opened 5 minutes for ventilation.

**Fig. 7:**
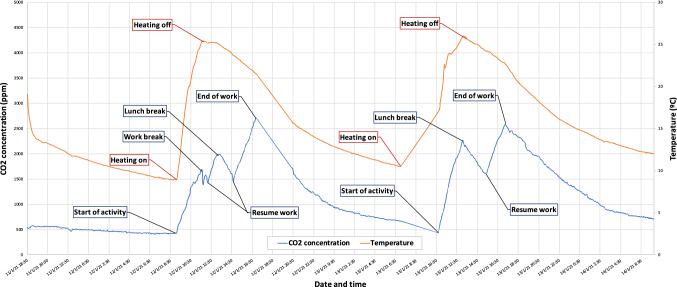
$CO_2$ measurement in a office taken over several days

The results obtained in the office, as shown in Fig. 7, emphasize that the $CO_2$ levels are reduced to low levels after during several hours while the office is empty, even with closed doors and windows. This confirms that the self-calibration of the nodes is possible, allowing us to calibrate in the same way classrooms when they are empty. During the breaks, when the office was empty but with the heating on, $CO_2$ levels decreased and then increased again when the activity was resumed. This shows that this type of heating does not affect the measurements taken. Also notice that after lunch time, while being the heating system off, the increase in $CO_2$ levels is similar to the ones when the heating system was on.

**Fig. 8:**
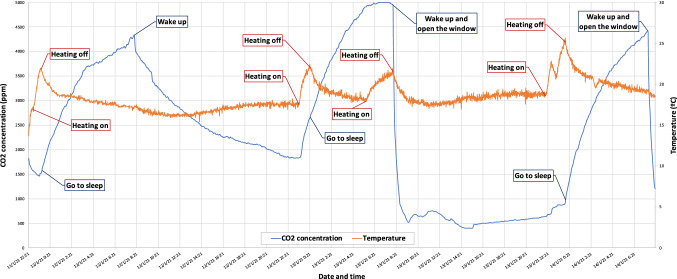
$CO_2$ measurement in a bedroom taken over several days

In the case of the bedroom, it can be seen in Fig. 8 that $CO_2$ levels are much higher than those observed in the office. However these levels start to increase when the electric heating radiator is switched on, despite the fact that anybody remains in the room, which means that this type of heating system affects the concentration of $CO_2$ in the environment. After the first night, the window was not opened for ventilation, so it can be seen how $CO_2$ levels slowly dropped to their initial levels. However, after the second and third night, when the window was opened for ventilation, the levels dropped to a minimum in a very short time (in the order of minutes), which demonstrates how ease and quick a closed space can be adequately ventilated.

### Measurements in the examination period

**Fig. 9:**
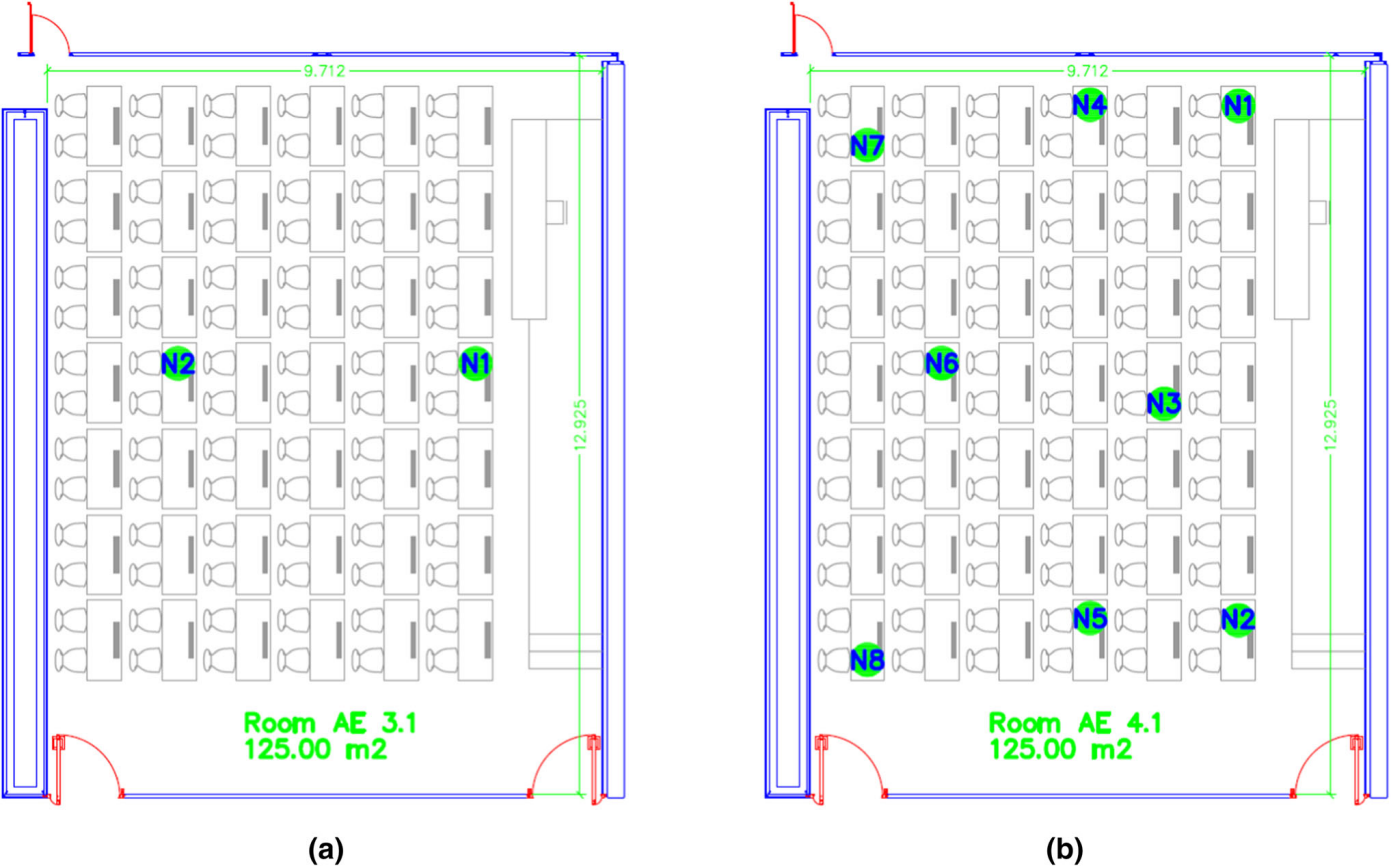
Location of the nodes in classroom 3.1 (**a**) and classroom 4.1 (**b**) at the ETSE

In order to test our system in a field evaluation, we have made some measurements during different exams in the examination period in December 2020 at room number 3.1 and January 2021 at room number 4.1 in the ETSE of the University of Valencia. Figure 9 shows the location of the nodes in the classroom 3.1 (Fig. 9a) and 4.1 (Fig. 9b).

A photograph can be seen during the measurements in Fig. 11. As these nodes are equipped with batteries, they can be located anywhere. Also, as shown in this figure, doors and windows are opened, ensuring a good ventilation, following the COVID-19 instructions.

**Fig. 10:**
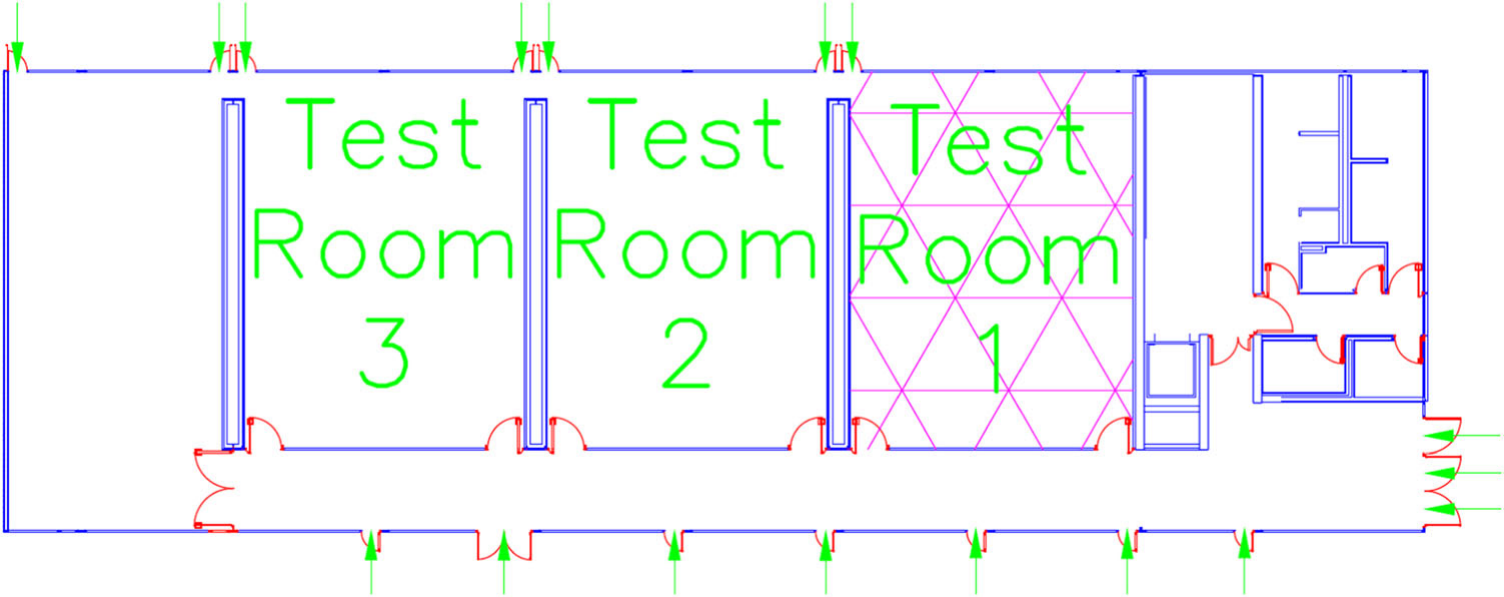
External air intakes in the building at the ETSE (green arrows show the external air input to the block 4 of the building)

Both classrooms are located in different buildings with identical distribution. Also, they have multitude of air inlets from the outside, as we can see in Fig. 10, marked with green arrows. These inlets promote a good ventilation inside the classroom, ensuring low levels of $CO_2$. The location of the classrooms inside the building is marked as Test Room 1.

**Fig. 11:**
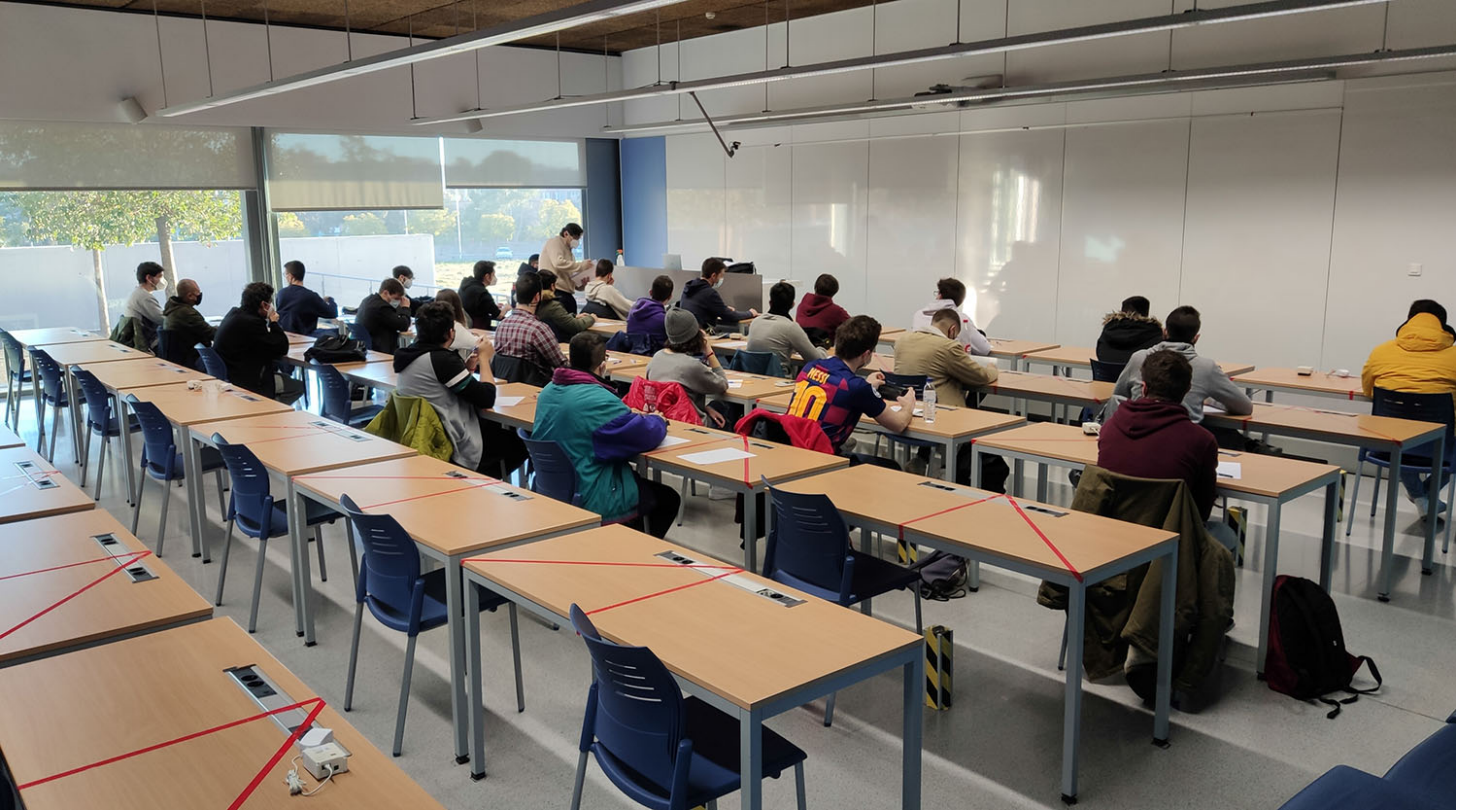
Photograph of the classroom 4.1 during the test

**Fig. 12:**
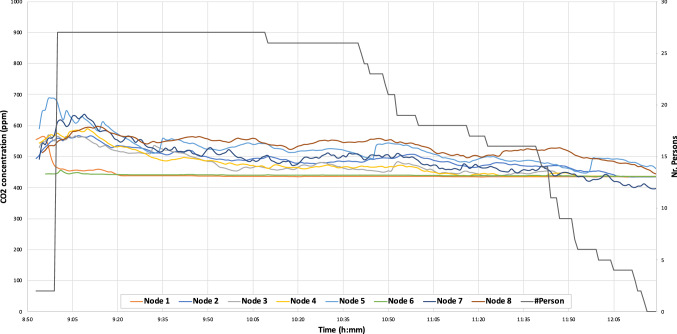
$CO_2$ measurement in room 4.1 at ETSE during an exam in January with 8 nodes vs. number of persons in the room (doors and windows were open)

**Fig. 13:**
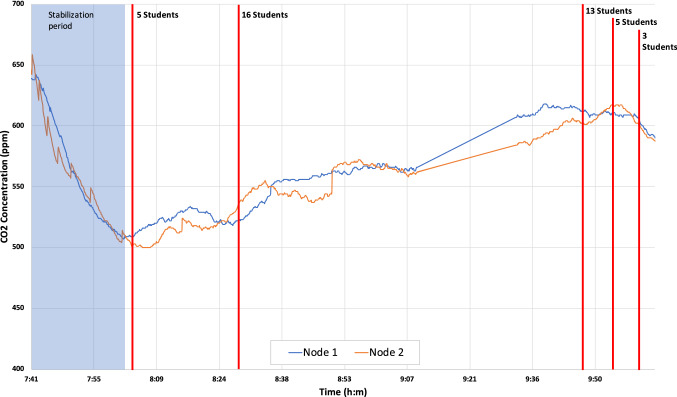
$CO_2$ measurement in a classroom during an exam in December with 2 nodes

In Fig. 11, we can see a photograph during an exam in January 2021. Figure 12 shows the time evolution of $CO_2$ concentration measured using eight different nodes. In both cases, the natural ventilation (due to open doors and windows) allows to keep the $CO_2$ concentration controlled, reducing the risks of COVID-19 contagions. These results show the correct behavior of the proposed system. In addition, Fig. 13 shows a line of the evolution of $CO_2$ concentration from two nodes during an exam in December 2020. In this figure, we can see a slight rise of the concentration, while the number of students increases. As the room is well ventilated, the $CO_2$ levels are not too high and under control.

### Spatial statistics

**Fig. 14:**
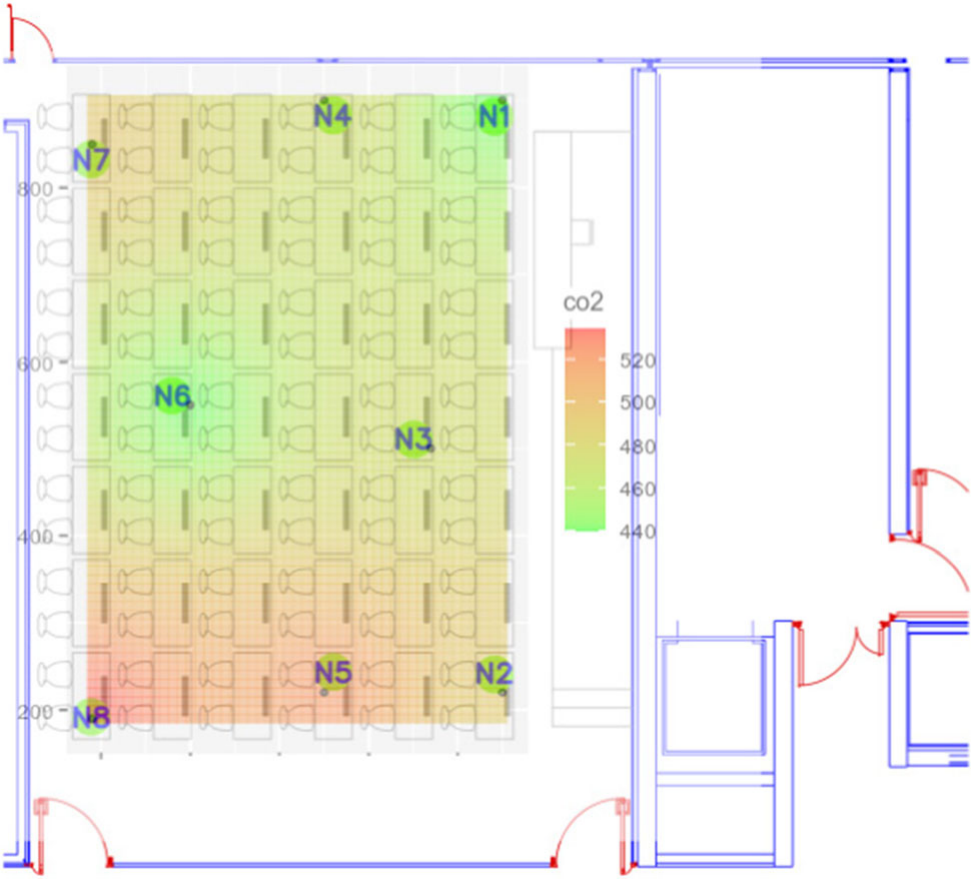
Spatial statistic representation of mean values of $CO_2$ concentration in room 4.1 at ETSE during an exam in January with 8 nodes

Figure 14 shows the spatial statistic study of the average of $CO_2$ concentration, using Kriging technique [8, 10, 21, 26] during the exam in January 2021. This method allows to predict (by means of statistical interpolation) the values inside the defined grid. In this case, we have used Ordinary Kriging [29] to compute the evolution of the spatial distribution of $CO_2$ concentration in the classroom. The video provided as supplementary material shows the time evolution per minute of the $CO_2$ concentration during the exam. It shows how well the class is ventilated during the exam

### Energy consumption performance evaluation

Now, we evaluate the energy consumption of the proposed nodes with two different configurations: (a) by using the MCU with the MH-Z19 and DHT22 sensors, together with a RGB led and an OLED screen and (b) by using both sensors without the RGB led and the OLED screen. To this end, we use a USB power-meter UM34C [25].

**Fig. 15:**
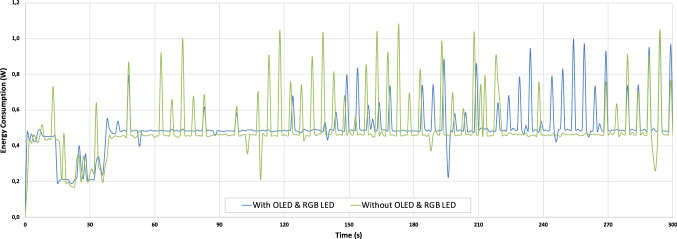
Energy consumption of the measurement and communication process in the node

Figure 15 shows the initialization of the node, with a starting sleep mode, and a measurement period in one node, with both configurations (a) in green color and (b) in blue color. We can observe from this figure that the consumption of the OLED screen and RGB led is very low, around 0.03W in average.

Also from Fig. 15, we can see different peaks corresponding to the measurement period of the MH-Z19 sensor, since the Infrared (IR) source of the NDIR switches on every 5 s during 400 ms. Therefore, we can conclude that these peaks correspond to the energy used to switch this IR lamp on. It must be noticed that these nodes have been designed to operate with 4 AA batteries for short-term measurement campaigns, in places where direct power supply is not possible. Therefore, these tests have been carried out to verify the duration of the nodes when measuring.

**Fig. 16:**
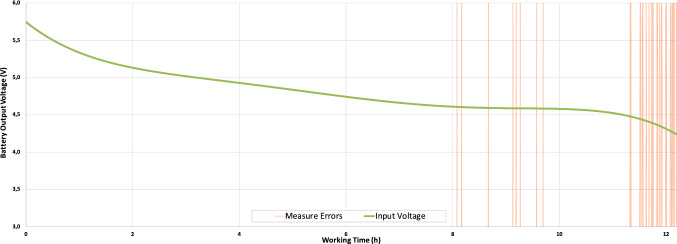
Operation and duration of the node with batteries

Energizer Max E91 type AA batteries [6] have been used and the node has been configured to take and send samples every 5 seconds with the display and RGB led on. Both the samples sent and the voltage supplied by the batteries have been monitored. Figure 16 shows the output voltage of the batteries and the errors in data readings. As it can be observed, errors start appearing sporadically after 8 hours of uninterrupted operation when the voltage drops to 4.6V and they continue working during 11 hours more, when the voltage drops to 4.5V. Finally the node stops working after more than 12 hours when the voltage drops below 4.2V.

## Conclusions

In this work we have developed a fully operative open-hardware and open-software fully operational IoT system and architecture to measure $$CO_2$$ concentration, temperature and relative humidity. This system is fully scalable and automatically upgradeable, thanks to the OTA updating function. The building cost of this system is low-cost, less than 100$.

This system has been tested in different environments: at work, at home and in class during different exams. The measurements taken shows full range operation (limited only by the sensor performance), allowing us to show perfectly the temporal evolution of the $CO_2$ concentration.

In order to study the spatio-temporal evolution of the $CO_2$ concentration measured in different situations, the architecture has been empowered with cloud-based processing capabilities to achieve Kriging technique to perform spatial interpolation of the metrics.

Finally, the energy consumption of the developed nodes has been also evaluated in each part of the circuit, lasting till 12 hours of continuous monitoring. As a future work, we are going to develop the node using a Fipy MCU which will allow 5G communication with these nodes. As this is a modular system that can be improved and upgraded (due to its openness). The use of other sensors such as PM3005 (for monitoring PM2.5 or PM10), TVOC and other gasses can be a matter of improvement by adding I2C, SPI or other interfaces to the sensing module. We also would like to explore ultra low power consumption real-time processors to extend the duration of the system with the same number of batteries.

## Supplementary Information

Below is the link to the electronic supplementary material.

Supplementary file1 (AVI 9065 KB)

## Abbreviations

TVOC: Total Volatile Organic Compounds
RH: Relative humidity
NDIR: Non-Dispersive InfraRed
UART: Universal asynchronous receiver-transmitter
ETSE: Escola Tècnica Superior d'Enginyeria (Engineering Technical School)
SARS-COV-2: Severe Acute Respiratory Syndrome COronaVirus 2
MCU: Micro-controller unit
EPROM: Erasable Programmable Read-Only Memory
IoT: Internet of Things
RPi: Raspberry Pi
Wi-Fi: Wireless Fidelity (IEEE 802.11)
OTA: Over the air
OLED: Organic light-emitting diode
RGB: Red-Green-Blue
COPD: Chronic Obstructive Pulmonary Disease
WSN: Wireless sensor network
LTE: Long term evolution
BLE: Bluetooth low energy

## Footnotes

1 https://github.com/jandrassy/ArduinoOTA
